# Barriers and unmet needs related to healthcare for American Indian and Alaska Native communities: improving access to specialty care and clinical trials

**DOI:** 10.3389/frhs.2025.1469501

**Published:** 2025-04-03

**Authors:** Donald Warne, Twyla Baker, Michael Burson, Allison Kelliher, Melissa Buffalo, Jonathan Baines, Jeremy Whalen, Michelle Archambault, Kimberly Jinnett, Shalini V. Mohan, Rebekah J. Fineday

**Affiliations:** ^1^ Johns Hopkins Bloomberg School of Public Health, and School of Nursing, Baltimore, MD, United States; ^2^ Nueta Hidatsa Sahnish College, New Town, ND, United States; ^3^ Sanford Roger Maris Cancer Center, Fargo, ND, United States; ^4^ American Indian Cancer Foundation, Minneapolis, MN, United States; ^5^ Mayo Clinic, Rochester, MN, United States; ^6^ Genentech, Inc., South San Francisco, CA, United States; ^7^ UCSF Institute for Health and Aging, San Francisco, CA, United States; ^8^ Sanford Health of Northern Minnesota, Bemidji, MN, United States

**Keywords:** American Indian/Alaska Native, healthcare access, healthcare barriers, health equity, health disparities, Indian Health Service

## Abstract

Substantial healthcare barriers, especially to specialty and cancer care, exist for American Indian and Alaska Native (AI/AN) individuals and communities at all levels. The unique history of AI/AN Tribal Nations and resulting policies, treaties, and relationships with the US government and federal agencies have created specific barriers to healthcare and clinical trial access for AI/AN peoples. Commonly, AI/AN peoples harbor a long-standing mistrust of the healthcare system based on lived and historical experiences. The intersection of various barriers to care for AI/AN communities results in health inequities, lack of representation in clinical research, and other disparities faced by historically marginalized and underrepresented peoples. AI/AN patients face unique barriers in their healthcare journey due to a disproportionate burden of life-threatening and chronic diseases, including many cancers. Identifying barriers specific to AI/AN peoples and improving access to high-quality care, with a focus on building on the strengths and capacities in each AI/AN community are vital to improving health equity. In this review, we describe patient, provider, and institutional barriers to healthcare, particularly specialty care and clinical research, for AI/AN peoples, with a focus on the Northern Plains AI communities. Examples and best practices to improve AI/AN patient access to health services, including screening and specialty care, as well as to clinical research, are provided. We emphasize the importance of longitudinal community-based partnerships and strength- and trust-based approaches as essential components of promoting equitable access to high-quality specialty care and recruitment and participation of AI/AN individuals and communities in clinical research.

## Introduction

American Indian and Alaska Native (AI/AN) communities face substantial barriers to healthcare, particularly specialty and cancer care. Improving access to high-quality care by identifying barriers to be addressed, focusing on strengths in AI/AN communities, and building sustainable relationships is vital to improving health equity. Healthcare barriers for AI/AN communities exist at the patient, provider, and institutional levels; a key component at all levels is a profound experience of inequity, racism, and marginalization experienced by AI/AN communities and individuals. Additionally, the challenges in the medical system include a shortage of AI/AN providers and omission of AI/AN community leaders and cultural practices. Barriers to healthcare in AI/AN communities stem from the unique history of colonization, public policies, and strained relationships with the US government (1, 2).

These healthcare barriers are especially concerning because AI/AN communities have a disproportionate burden of life-threatening and chronic diseases, including cardiovascular disease, COVID-19, type 2 diabetes, stroke, and certain types of cancer (3, 4). Morbidity and mortality rates in AI/AN peoples are higher than in the total population (all races) (5). AI/AN individuals also experience greater overall mortality rates and higher rates of death from heart disease and cancer than Black/African American, Hispanic/Latino, and White individuals (6). From 2014 to 2018, the overall cancer incidence was 2% higher in AI/AN individuals than White individuals, and mortality rates for infection-related cancers and kidney cancers were approximately two-fold higher in AI/AN individuals than White individuals (4). Furthermore, the COVID-19 pandemic disproportionately affected AI/AN communities and revealed existing health disparities (7). Historical loss of land, cultural disruption, and lack of access to healthcare, housing, healthy food, and educational and employment opportunities all contribute to health disparities in AI/AN communities (8–11).

The disproportionate burden of chronic diseases and health disparities in AI/AN communities makes the especially low rate of clinical trial enrollment for AI/AN peoples troubling (12). Tribal Nations have a unique history with the US government that contributes to specific barriers for historically underrepresented and underserved groups at the level of the patient, provider, and institution (13). Structural racism and implicit bias contribute to limited awareness, logistical barriers, mistrust of the healthcare system, and lack of culturally safe care (14, 15). In addition to the systemic and social barriers, mistrust between AI/AN communities and investigators due to past unethical conduct and lack of understanding of the community has exacerbated low enrollment numbers (16–18). Clinical trials are considered the gold standard for researching new healthcare treatments (19), and non-representative research limits the generalizability of study results and can hinder understanding of benefits and risks of therapies across populations (20, 21). Non-representative trials limit access to potentially lifesaving care, which can further aggravate existing healthcare inequities. For example, at the start of the COVID-19 pandemic, there were no approved treatments, and access to some treatments was limited to patients enrolled in clinical trials (22).

The objective of this article is to improve awareness of the most relevant and actionable barriers to healthcare, including specialty care, and clinical trials for AI/AN individuals, with a focus on Northern Plains AI communities based on the lived experiences and expertise of several authors. The strengths within AI/AN communities to overcome barriers and the necessity of building trust and relationships with AI/AN communities are emphasized. Best practices to improve patient access to clinical research and greater health equity are addressed.

## Historical context

A thorough and truthful understanding of the history of AI/AN peoples is important to understand the bases of health disparities and better address the healthcare needs of AI/AN communities. Prior to colonization, AI/AN cultures had unique and distinct systems of reciprocity as well as land- and place-based healing practices that honored the interrelatedness of their health and lifestyles. A history of colonization, public policy, and systemic racism has contributed to educational, health, and economic inequities for AI/AN communities in the US (23).

### Challenges surrounding colonialism and tribal sovereignty

The diversity of Tribes in the US is extensive, with distinct cultures, languages, and political and sociodemographic history. There are 574 federally recognized Tribal Nations and hundreds of others seeking state and federal recognition (24). Tribal governments are sovereign entities; some Tribes have a land base on a reservation and some do not. There are approximately 326 federally recognized American Indian reservations, with most governed by a single Tribal Nation, and others governed by two or more Tribes (24).

In health research, AI/AN populations are most often considered a homogenous racial group; however, these populations are made up of diverse and culturally distinct groups. Tribal Nations, even those that live in close proximity to one another, may have different traditions, cultures, languages, and norms due to the history of relocation policies. By grouping AI/AN populations together, researchers may miss key health trends in a specific Tribe/sub-population or overestimate the effect of clinical trials on these communities (25).

AI/AN peoples have cultural and healing practices that have been built in close relationship with the earth over generations. Extractive processes (e.g., mining, quarrying), loss of natural resources, and relocation have contributed to health inequities (27). Throughout US history, many Tribes were forced onto land with fewer natural resources (27). Currently, Tribal Nations disproportionally face increased climate vulnerability and pollution and diminished economic value of their lands (27). Reservation territories endure more severe heat and less precipitation and are at higher risk for land degradation. Only a small proportion of reservations have oil- or gas-producing wells or are located on lands with significant economic and mineral value (27). Despite geographic and resource limitations, many Tribal Nations effectively exercise sovereignty, practice traditional cultures, and preserve traditional languages.

### Public policy practices detrimental to the health and wellbeing of AI/AN people

Between 1819 and the 1970s, the US government established and supported Indian boarding schools across the country, with the objective of assimilating AI/AN children into mainstream American culture (28). The children who attended boarding schools were often forcibly separated from their family and reservations, stripped of their native language and culture, and subjected to a systematic, militarized environment where corporal punishment was often a means of enforcing rules. Accounts of physical, sexual, and emotional abuse; disease; malnourishment; overcrowding; and lack of healthcare in these institutions are well documented (29). Survivors often developed both mental and physical health problems, including posttraumatic stress disorder, substance use disorder, and cardiovascular disease. The experiences of these children were passed on to future generations through historical trauma, leading to physical and psychological problems that persist (29).

The Family Planning Services and Population Research Act of 1970 led to the forced sterilization of approximately 25% of AI/AN women of childbearing age, including procedures done under duress or without the women's knowledge or consent (30). Even after the passing of the Indian Health Care Improvement Act in 1976 (26), which gave Tribes the right to manage or control their own healthcare programs, the substantial negative effects of forced sterilization and other discriminatory policies had created fundamental mistrust in the healthcare system.

### Healthcare challenges on reservations

The dispossession of AI/AN peoples of their Indigenous lands and their subsequent removal to reservations has exacerbated the disparities between them and the general US population. Compared with the general US population, AI/AN households on reservations tend to be larger and are more likely to live in crowded dwellings, report lower median household income, and have houses without indoor plumbing (31). Healthcare challenges are worsened by chronic underfunding of the Indian Health Service (IHS), which is frequently focused on isolated systems and responding to chronic diseases instead of basic public health infrastructure (32, 33). Formed in 1955, the IHS is responsible for providing health services to AI/AN individuals and is the principal healthcare provider and health advocate for AI/AN peoples (34, 35). However, the IHS is not a health insurance program, such as Medicare or Medicaid, and only provides care at its federal hospitals and clinics (36). Insufficient funding, geographic isolation, limited availability of clinicians, and limited access to healthcare and services in the IHS are examples of infrastructure challenges to AI/AN peoples' access to health services (37) that can be tied to dispossession of Native lands. Additionally, the loss of food sovereignty and traditional food systems resulted in dependence on federal food programs, such as the Food Distribution Program on Indian Reservations (FDPIR) (9), a US Department of Agriculture program that historically provided unhealthy food options. FDPIR and other food programs have been substantial factors in declining health due to poor nutrition (38).

### Future optimism

Despite being subjected to colonialism, loss of land, and policies such as assimilation, relocation, and Tribal termination, many AI/AN communities are thriving in terms of cultural resilience and strength. In the words of one author (R. F.), “While [there were] many attempts at extermination, we are still here, doing the best we can living in two worlds, including the one that tried to destroy us.” Wide-scale community efforts are being made to retain cultures and languages among Tribes, and intergenerational communication and connection between Tribal Elders, youth, and the Earth are key to creating healthy life philosophies (39). Tribally centered leadership programs that are specifically designed for AI/AN youth instill purpose and community and are important tools for developing healthy lifestyles (40).

## Barriers to care

**Figure 1:**
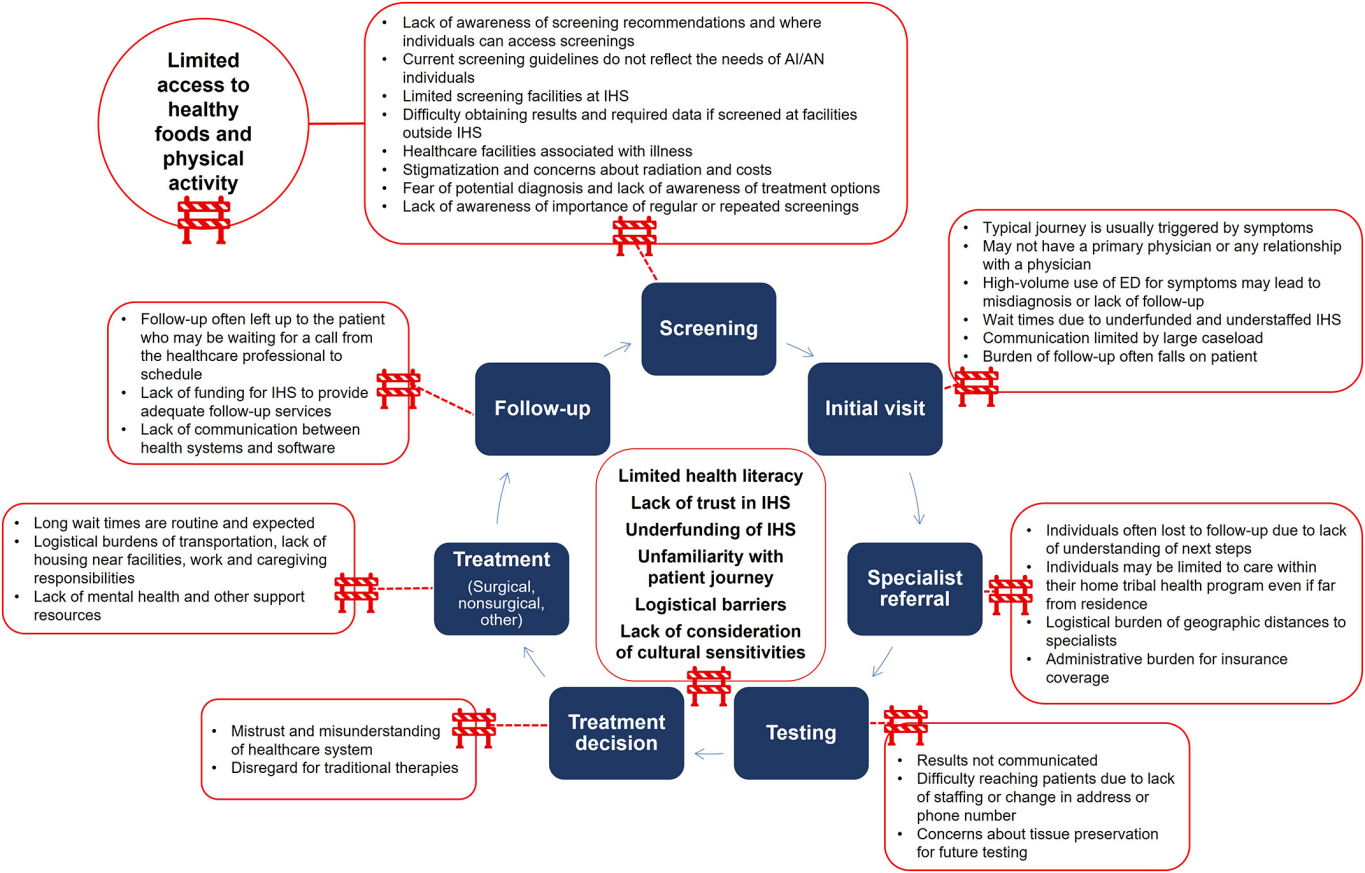
Barriers along the patient journey for AI/AN individuals. AI/AN, American Indian and Alaska Native; ED, emergency department; IHS, Indian Health Service.

Increased access for AI/AN individuals to specialty care and clinical research begins with access to basic health services and screening tests and subsequent establishment of trust between researchers and participants (16). AI/AN populations face many barriers to healthcare in their patient journey, including low health literacy and barriers to clinical trial enrollment (Figure 1). These barriers may differ between Tribal communities due to cultural differences, geographic location, and Tribal resources. Here, we will focus on barriers affecting AI/AN communities more broadly and those specific to Tribes in the Northern Plains.

### Patient-level barriers

Patient-level barriers to health begin long before a person sees a healthcare professional. AI/AN communities are disproportionately impacted by food insecurity, which is associated with poorer health outcomes (41, 42). These communities have limited choices and often rely on highly processed, obesogenic, and sodium- and sugar-rich foods (9). Traditional, natural, and healthy food preparation has been replaced by pan or deep frying. Although food programs such as the US Department of Agriculture Department of Defense Fresh Fruit and Vegetable Program allows Tribal organizations to purchase domestically grown and produced fresh fruits and vegetables, the FDPIR often does not have fresh vegetables or fruits listed on its available food list (43). Federal programs that promote poor nutrition are often rooted in unjust policies designed to eradicate AI/AN peoples and cultures (9). In addition to nutritional barriers to health, AI/AN populations lack opportunities to safely recreate and have among the lowest levels of physical activity in the US (44). Physical activity and exercise are key aspects of prevention for diabetes, obesity, hypertension, and cardiovascular disease (45). Intergenerational interruption of subsistence and land-based lifeways have denied AI/AN peoples safe spaces to be in and practice traditional games and gatherings, further exacerbating disparities (46, 47). Supporting health equity through a public health lens before a patient becomes ill by addressing systemic upstream issues can be a fundamental step toward positive health changes in AI/AN communities.

Cultural shifts forced by the federal government led to health inequities including criminalizing traditional practices of prayer and ceremonies through the Code of Indian Offenses, in which courts were held to adjudicate the so called “offenses.” (48) The governing rules of the courts included the restriction of traditional tobacco use for medicinal or ceremonial purposes, such as healing ceremonies, which resulted in increased access to and use of commercial tobacco, further worsening health outcomes. Unfortunately, AI/AN communities have been specifically targeted by marketing from the tobacco industry (49). Sovereign Tribal Nations are also not subject to state and local policies, including tobacco taxes (50, 51), which have decreased smoking and second-hand smoke exposure in the general population. As a result, AI/AN communities have had the highest commercial tobacco use among all races and ethnicities since 1978, and smoking prevalence is highest in the Northern Plains region (52).

Indigenous communities understandably retain a deep mistrust of the healthcare system. Historically, medical research has exploited AI/AN populations and ignored Indigenous values (17, 18, 53). The history of forced medical experimentation conducted on children and adults, including the involuntary sterilization of women, persists in the living memory and experiences of AI/AN communities. The long-term, compulsory boarding schools for Indigenous children implemented by the Bureau of Indian Affairs were sites of abuse, neglect, and involuntary experimentation on drugs and vaccines (29, 54). Additionally, negative experiences with the IHS for a patient's own needs or that of family members are common and include long wait times to schedule and receive care due to lack of IHS providers (55), along with poor care and outcomes.

Patient perceptions and fears further exacerbate challenges in obtaining optimal healthcare. For many AI/AN individuals, healthcare facilities may be associated with illness and treatment rather than preventive care and screening. Based on the experience of one author (R. F.), patients are sometimes unwilling to travel, despite the higher level of care provided at hospitals, due to negative connotations associated with these facilities, including the impression that the clinic is where people get sick and die. AI/AN individuals may also avoid preventive screening due to being asymptomatic, lack of family history of disease, perceived stigmatization toward tests, concerns about the safety or costs of tests, fear of potential diagnosis, and lack of awareness of treatment options. Preventive care is underused by AI/AN individuals, who are less likely than White individuals to be up to date on cancer screening tests (56, 57). These barriers to preventive and cancer screenings specific to AI/AN peoples and communities may delay diagnosis and limit opportunities to participate in clinical trials.

Inadequate funding of the IHS contributes to inadequately serving the health needs of diverse populations that are often distributed over large distances (55). Lack of transportation is a barrier experienced by AI/AN peoples attempting to access quality healthcare (58). Geographic distances lead to long travel times, and may be notable for specialist care often required due to the high burden of complex chronic diseases. For example, AI/AN patients have been shown to have the longest travel times of any other racial or ethnic group for neuro-ophthalmology specialty care (59). Long distances from healthcare facilities may be complicated by winter weather conditions, which are of particular concern to peoples in the Northern Plains (60); in addition, many remote Native communities in Alaska are only accessible by airplane or boat (61). According to one author (R. F.), patients report that even when obtaining transportation through Tribal resources, access can be problematic; these rides may not arrive on time due to the logistical difficulty of multiple patient and passenger pickups.

Scheduled visits that require frequent travel to and from healthcare facilities over long distances and a lack of meals or housing for individuals while undergoing treatment may limit care and participation in clinical trials. Deficiencies in food programs (described earlier in this section and in the Historical Context section) may deny patients access to the quality food and meal preparation needed to travel long distances. Additional factors that may potentially contribute to lack of access include the business hours at the facilities (including limited 24-h care), lack of job flexibility to leave work for multiple appointments, and the responsibility of caring for children or others in the community. Lack of childcare may result in children having to accompany their adult caregiver to the hospital or medical clinic.

Due to the social, emotional, and historical differences from White Americans, AI/AN patients may be less familiar with the patient journey and healthcare system. Some individuals may not receive adequate guidance or education to fully understand the steps that follow screening tests. For example, a multilevel, multicomponent interventional trial reported a large discordance between the prevalence of self-reported comorbidities and measured risk factors of cardiovascular disease (62). AI/AN individuals' lack of resources contributes to an inadequate capacity to arrange the necessary next steps to access follow-up care. Follow-up is often left to the individual patient, who may not be fully aware of the next steps for a specialist visit.

Administrative burdens may also contribute to lack of access. Although all enrolled Tribal members are guaranteed healthcare through the IHS, it is not a health insurance provider (36). A lack of understanding of the IHS system among patients, providers, and healthcare systems may lead to billing errors, which are usually to the detriment of the patient. For AI/AN individuals who do not have health insurance, IHS Purchased and Referred Care funds are used to pay for private sector referrals (36); however, referrals may be limited due to underfunding of the program (63). AI/AN individuals may have access to health insurance through public programs such as Medicaid or Medicare or through the Affordable Care Act Marketplace; however, administrative burdens related to understanding eligibility and enrolling in the plans may limit enrollment even among those who are eligible (64, 65). Additionally, AI/AN individuals may feel that they should not have to apply for health insurance because health services were promised to Tribal Nations by the federal government as part of the proper care and protection guaranteed in treaties (1).

### Provider-level barriers

High vacancy rates of healthcare professionals in IHS facilities negatively affect patient access and quality and continuity of care (66). Limitations to filling vacancies include remote locations, limited housing for providers, and inability to match market salaries. Additionally, AI/AN peoples are substantially underrepresented in the workforce of physicians, practitioners, researchers, and other healthcare providers. Only 1.1% of enrolled US medical students for 2023–2024 are AI/AN alone or in combination with another race/ethnicity (67). Understaffed IHS facilities can result in large caseloads for healthcare professionals, which may prevent adequate communication with patients. Understaffing along with high turnover rates can reduce the continuity of care and trust in these facilities.

Another barrier related to providers is the use of one-size-fits-all disease screening guidelines that may be inadequate for AI/AN populations (68), along with lack of awareness on where patients can access follow-up testing and treatment. Typically, guidelines for screening were created without considering the needs of and disease incidence in AI/AN patients or other underserved communities (69). Rates of cancer vary between AI/AN and White populations, including an overall higher cancer incidence in AI/AN individuals than White individuals, and two-fold higher mortality rates for infection-related cancers and kidney cancers (4). AI/AN individuals have a higher rate of liver cancer than White individuals in all regions, and higher rates of colorectal, kidney, and stomach cancers than White individuals in almost all regions (70). Rates also differ between AI/AN regional populations, for example, overall cancer incidence is more than twice as high in AI/AN individuals living in the Southern Plains than in the Southwest region (70). In Alaska, stomach cancer is the fourth most common cancer for AI/AN communities and the sixth most common for those living in the Southwest region but is not among the top ten in other regions within the United States (70). Additionally, AI/AN individuals living in the Northern Plains have a five-times higher incidence of lung cancer than those living in the Southwest region (71).

Finally, healthcare professionals are susceptible to implicit bias, the negative and sometimes unconscious attitude someone holds against a specific group. A systematic review found a negative correlation between the level of implicit bias and indicators of quality of care (72). Additionally, medicine is frequently viewed from an authoritarian, patriarchal perspective, and medical residents often prefer paternalistic decision-making for patients (73). Providers and researchers may be unaware of the historical traumas that play a large role in the mental and physical health of AI/AN communities and lack cultural sensitivity to embrace the values and traditions held by tribal members regarding their health.

### Institutional-level barriers

Tribal Nations were promised healthcare services by the federal government as part of the proper care and protection guaranteed in treaties (1). However, a crucial barrier to healthcare faced by AI/AN populations is the long-term underfunding of the IHS (1, 37, 74), which is a contributing factor to health disparities in AI/AN populations (1). The US government allocates smaller proportions of money per capita to the IHS compared with all other federally funded healthcare programs: in 2017, IHS per capita spending was $4,708 compared with $8,109 for Medicaid, $13,185 for Medicare, $10,692 for the Veterans Health Administration, and $8,600 for federal prisoners (75). In addition, lack of Medicaid expansion in some states limits access to healthcare and potential clinical trial participation for AI/AN individuals (76). The lack of infrastructure and financial support from federal institutions creates significant barriers for AI/AN populations.

IHS is the federal healthcare provider for AI/AN individuals, but its facilities on reservations are typically not owned or operated by local Tribal Nations (36). Tribal health units were created after the Indian Self-Determination and Education Assistance Act, which allowed Tribes to manage their own healthcare services that had previously been under federal control (77). While there are many advantages to Tribal health units, including the increased ability to provide culturally appropriate care and a focus on prevention and opportunity for local partnerships, there are still systemic barriers between federal and locally run health centers that impede patient care. Patients who need specialty care may face delays and logistical barriers due to a lack of communication between Tribal health units and IHS and requirements for care at the home Tribal health unit, which may not be based on a patient's geographic location.

Another institutional-level barrier is the tendency of many industry sponsors to use the same well-known investigators at research and academic centers for clinical research, which generally do not provide care to underserved and diverse communities. In 2022, the American Society of Clinical Oncology and Association of Community Cancer Centers released a joint research statement regarding the need to increase racial and ethnic diversity in cancer clinical trials (78). One of their six overarching recommendations is for clinical trial sponsors and researchers to design and implement trials with a focus on reducing barriers. This involves partnering with community leaders, ensuring clinics have access to a diverse portfolio of clinical trials, and developing policies to increase the number of research sites. More focus and funding aimed at reducing barriers to care may allow for a wider and more diverse population of patients to be enrolled in clinical trials.

## Overcoming barriers to access to specialty care and clinical research

Steps to overcoming barriers should be focused on the leadership, voices, and strengths in AI/AN communities as well as Tribal communities being more open to receiving technical assistance from non-Tribal entities. The importance of improving access to specialty care, clinical research, and innovative therapies for AI/AN populations with a goal of improving health outcomes cannot be overstated. It should be emphasized that each AI/AN community is unique, with their own history, culture, and language; therefore, removing or reducing specific barriers may have different results among individual Tribal Nations.

### Decreasing patient-level barriers

**Figure 2:**
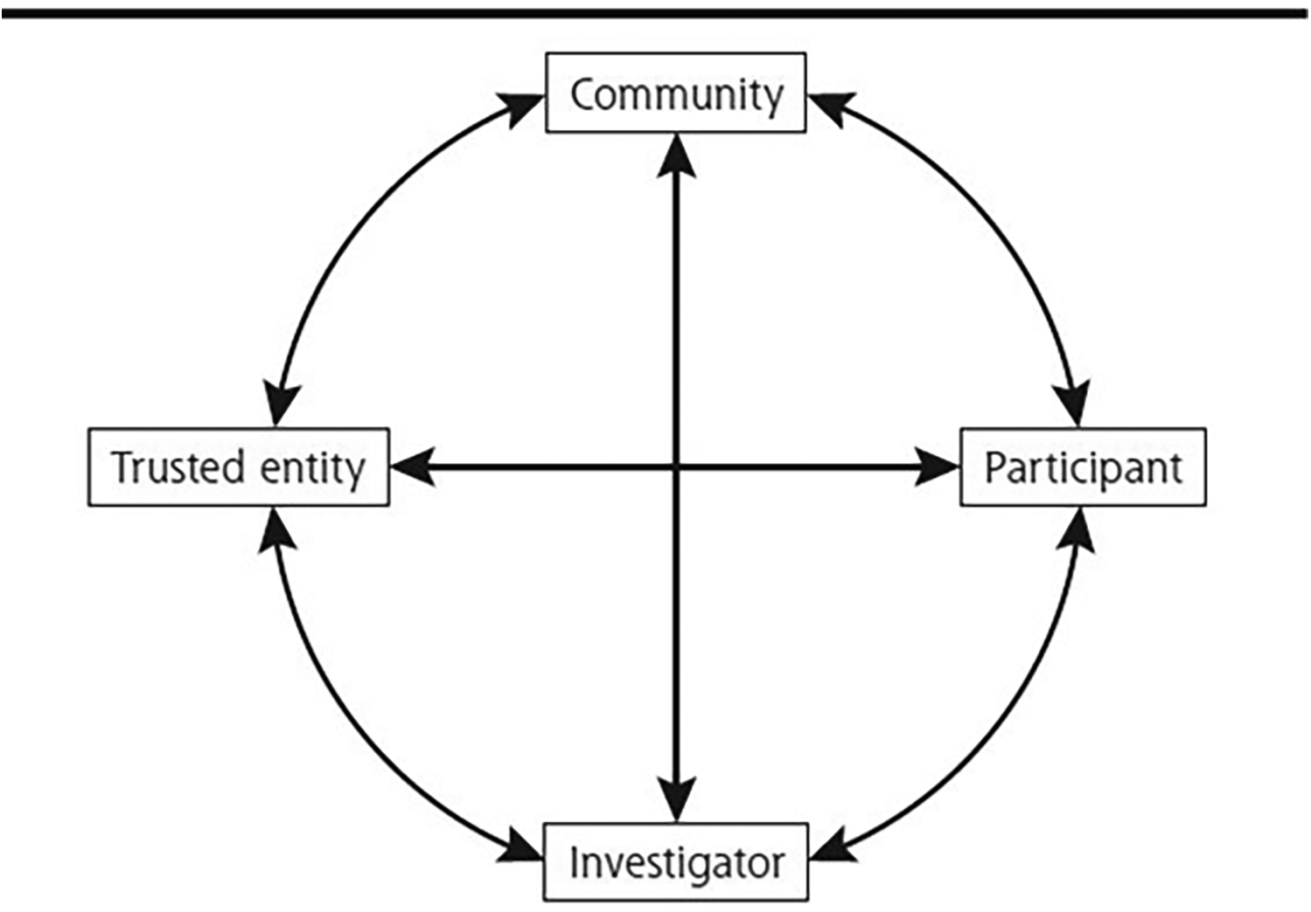
Circle of trust. Reproduced with permission from “The circle of trust” by Arch G. Mainous, Allison Kelliher and Donald Warn. January 2023, 21(1)54–56. © 2023 Annals of Family Medicine, Inc. All Rights Reserved.

Effective communication and education are essential for decreasing patient-level barriers and should involve Tribal leaders and community stakeholders. It is important that patients feel that their cultural history and present circumstances are being observed and respected; the Circle of Trust approach is one way to achieve this (16). The Circle of Trust is a model designed to improve the recruitment of AI/AN populations that was developed by AI/AN investigators who conduct research in AI/AN communities (16). The circle model emphasizes the importance of community perspective and involvement with bidirectional arrows between the four quadrants of trial participant, community, trusted entity, and investigator (Figure 2). Community plays a central role in AI/AN cultures, and community-based participatory research includes the community as an *equal* partner with the funding agency and research institution (79). Use of trusted messengers is a strategy that has been used to provide accurate information to encourage behavior change related to the COVID-19 pandemic (80). Furthermore, patient trust has been shown to be a vital part of the patient-provider relationship (81).

A storytelling approach for education and communication, including patient materials and tool kits, is a useful strategy for engaging with AI/AN communities and promoting participation in health research (82). Furthermore, all patient educational and clinical trial materials should be accessible and understandable to patients and their families and be designed to be culturally appropriate and build trust in the healthcare and research arenas. Important suggestions include the use of familiar, cultural terminology and imagery and to frame messages in the appropriate cultural context. For example, commercial tobacco should be clearly differentiated from the traditional tobacco used in important ceremonial practices. It is also important that research results be communicated with patients, Tribal leadership, and the community in a format that is culturally appropriate and understandable; the community should be acknowledged and credited for their support, and how the study benefits the community should be emphasized.

The use of patient navigators to assist with scheduling, administrative tasks, follow-up, and other aspects of the patient journey may help ease some of the logistical burden on patients. Cultural navigators who have a shared history and learned experiences of Tribal Nations and are aware of and have access to community resources, including events, Tribal Elders, and spiritual advisors could be another source of support for AI/AN patients. Ideally, these cultural navigators would be from the same local Tribal Nation as the patients.

Part of gaining the AI/AN peoples' trust in the healthcare system is through the incorporation of traditional medicines and awareness of the value of Indigenous culture in supporting a patient's healing. Many articles about Indigenous medicine are written from non-AI/AN perspectives, which can often miss important cultural nuances (83). Incorporating traditional healing practices, including healing ceremonies, into Western practice may not only benefit the patients but also the providers. Traditional cultural healing practices are varied and include physical modalities, botanical and spiritual practices, and ceremonies (84). Traditional AI/AN healing practices already have a presence in modern Western medical practice. For example, osteopathy has grown out of the practices of physical manipulation that are rooted in the traditional and ongoing practices of the Oto, Shawnee, and Eastern Band Cherokee Tribes (perhaps in addition to others) (85, 86). Many botanical and herbalism practices are based in plant medicines of the unceded lands of North America (87). Additionally, practices such as healing touch, known to diminish pain and expedite hospital discharge with reduced medications, are also sourced from holistic perspectives with Indigenous Native American roots (88). Many Tribes incorporate traditional healers, including hands-on practitioners, counselors, doulas and birth attendants, community health workers, behavioral health aids, family home visitors, Tribal Doctors, and Traditional Healers, in their health systems.

Traditional AI/AN practices are typically unappreciated by conventionally trained health practitioners (89). Recognizing the role of spirituality in health can be uncomfortable for some healthcare providers (91). Ceremonies play an important role in overall wellbeing for AI/AN peoples, with community and individual participation contributing to overall healing energy (90). For example, making smudging practices (ie, the burning of one of more medicines, such as sage or cedar, gathered from the earth) accessible in a clinic or hospital by providing a bundle of sage or a dedicated space may provide comfort to patients and their families (92, 93). Spiritual wellness and balance, including methodologies to remedy imbalance, are essential for health of many AI/AN individuals (90). The inclusion of factors that are greater than the individual, the community, and the family is also an important consideration for AI/AN health. This includes the person's relationship with the land and environment. Traditional healing approaches offer a unique model that holds tremendous promise to promote wellness and balance beyond the individual to provide collective benefit. To live in harmony with each other and the Earth has direct meaning and purpose, with benefits beyond singular outcomes (90).

Training programs are evolving to incorporate these valuable practices. This includes the University of New Mexico Public Health and General Preventive Medicine Residency, which has begun implementing a culturally based, traditional healing curriculum (93). Residents demonstrate skills in advising patients and promoting cultural sensitivity and knowledge of culturally based healing practices (93). The curriculum includes learning from traditional healers and participating in practices of healing. This residency curriculum produces physicians who are well trained in approaching patient care and population health and have knowledge of culturally based health practices to facilitate the health of patients and their communities. Alaska Pacific University also offers training for Traditional Healers who work in the clinical setting (94). Patient outcomes may likely improve when AI/AN peoples receive care provided by AI/AN peoples in a setting that promotes the basic tenets of health and balance by including cultural practices and healers and gives patients the space to incorporate traditional medicines.

Logistical and financial support at institutional and Tribal levels could also offer patients better access to care. Transportation should be consistent and reliable and allow for a support person to travel with the patient, with understanding and timely rescheduling if lack of transportation leads to missed appointments. As patients may travel long distances to appointments, support and/or stipends for lodging, meals, childcare, caregivers, and caregiver transitions could decrease patient healthcare barriers. Some Tribal Nations provide limited financial support that may include travel and lodging for short-term medical emergencies (95); potential partnerships between counties, hospitals, and clinical trials may remove some barriers around logistical support for patients. Flexibility in timing of site visits and telemedicine options, with sufficient infrastructure and focus on the patient-provider relationship (81), can reduce a patient's emotional stress during a study. Allowing tours of the facility and discussion of the patient journey may also ease any pre-trial misconceptions. Additionally, seeing AI/AN patients in their own communities can allow providers to gain a better understanding of the community itself and any existing and potential logistical barriers.

### Decreasing provider- and study-level barriers

A strengths-based approach to healthcare research involving Indigenous populations and the education of healthcare professionals is a promising method to improve the health and wellness of AI/AN individuals (96–98). Healthcare is dominated by Western, allopathic practices, and the approach to health disparities in Indigenous communities commonly starts from a deficit-based perspective that focuses on the problems in communities, whereas strength-based approaches have been developed based on Indigenous perspectives on the relationships between individuals and communities, as well as the strengths and capacities of Indigenous populations (96, 97). Focusing on positive attributes, relationships, restoration of self-determination, and structural determinants of the health of Indigenous communities has been recommended as a strength-based strategy for the education of the healthcare community (97). Elevating Indigenous experts to lead policy initiatives and research projects is crucial to achieving health equity and improving outcomes. In addition, integrating traditional treatments and practices through Tribal Elders, spiritual advisors, or Traditional Healers and using sounds that are familiar and therapeutic as well as aromas and imagery to facilitate welcoming clinical environments may help to promote health equity. Based on the experience of one author (T. B.), healthcare systems that implement avenues for access to traditional treatments and practices see benefits in the form of improved relationships and dismantling of barriers to care for AI/AN populations. According to another author (R. F.), incorporating imagery and terminology familiar to AI/AN patients into visual and written educational items in clinics and hospitals creates a more welcoming environment that allows patients to feel more comfortable and relaxed. Additionally, asking AI/AN patients what they would like as part of their healing time in the hospital (e.g., offering to burn sage) and supporting their requests may help build trust and allow patients to incorporate their cultural and traditional practices.

**Figure 3:**
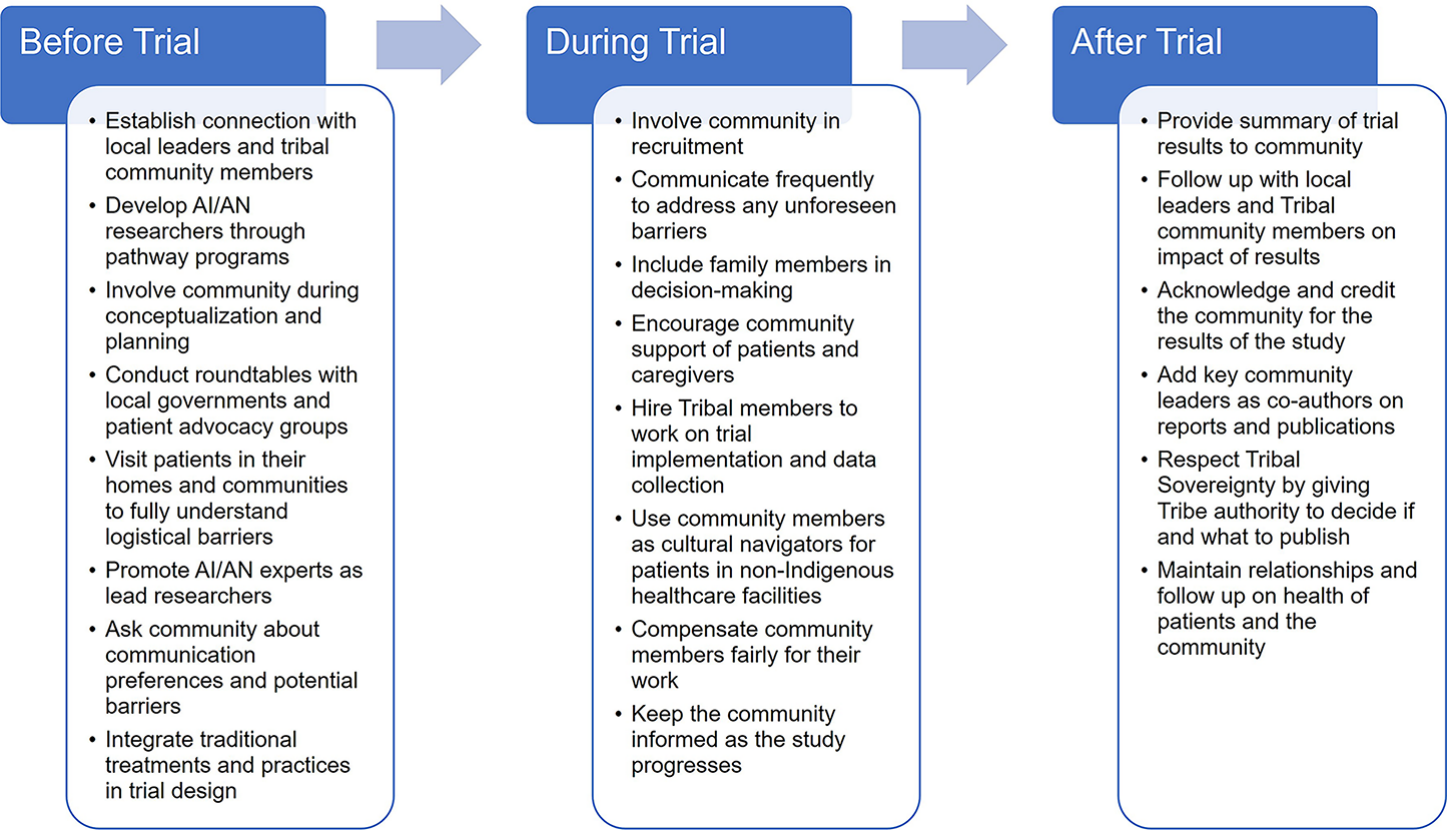
Opportunities for Tribal community involvement during all phases of clinical trials. AI/AN, American Indian and Alaska Native.

Establishing connections with local leaders and tribal community members may be a key path toward improving AI/AN health equity. Providers should be aware of and value cultural differences to fit the cultural context of AI/AN communities and use respectful and culturally appropriate language (99). While providers, pharmacists, and researchers who are serving AI/AN communities are helped by broad guidelines such as this, it is invaluable that they take time to know and understand their local Tribe's customs and cultural identity and communicate with Tribal leadership and Elders to intentionally include Indigenous perspectives and priorities in their practice. Healthcare providers should ask questions about items and ceremonies they see but do not understand. They should also ask the community to identify their wants, interests, and concerns and how they would like a particular topic addressed. This authentic community-based approach is essential to building trust and requires time for partnership building (100). In addition to working with Tribal leadership and Tribal Elders, healthcare providers should interact with local governments and advocacy groups through roundtables to focus on working in partnership to address specific challenges and needs and find solutions. Community leadership and participation before, during, and after a clinical trial is essential (Figure 3).

Clinician recommendations can be a key factor for patients considering participation in a clinical trial (101). Repeated use of the same large academic sites and well-known investigators, often located in urban areas, for clinical research can limit the awareness and participation of physicians in rural or community settings who provide care to underserved and diverse communities (102). Increased research at rural and community-based centers is an important part of increasing clinical trial diversity. In addition to widening research site selection, implicit bias based on stereotypes and structural racism, which may limit which patients are offered clinical trial participation, should be addressed. The screening process for evaluating eligible individuals should be objective to ensure an equitable process. There are several examples of program centers who specialize in partnering with indigenous rural communities that are creating positive connections between the AI/AN community and clinicians. One is the John Hopkins Center for Indigenous Health with a total reach of 170 tribal communities across 28 states (103). The center focuses on a number of areas including community engagement, diabetes and obesity prevention, infection disease prevention, mental health promotion, and COVID-19 response and prevention. By applying an emphasis on co-learning and flexibility, the center was able to spearhead studies investigating the impact of COVID-19 on the AI/AN research workforce (104) and food security (105). Sanford Health, the largest nonprofit rural US healthcare provider, has multiple programs focused on expanding healthcare access and improving outcomes for patients in rural areas, including medical flights for rapid access to specialty care, mobile mammography clinics, virtual consultations, integrated health therapists in primary care clinics and oncology departments, participation in over 400 clinical trials concentrating on recruitment of patients in rural populations, and community outreach to address food insecurity and reinforce healthy behaviors (106). Another example is the Avera Research Institute, which focuses on population health and the integration of clinical research studies in the health system, particularly surrounding AI/AN health. Using culturally appropriate, accessible approaches, the collaborative team was able to demonstrate the feasibility and efficacy of web-based mammogram interventions to promote breast cancer screening in an AI/AN community in the Northern Plains (107).

**Figure 4:**
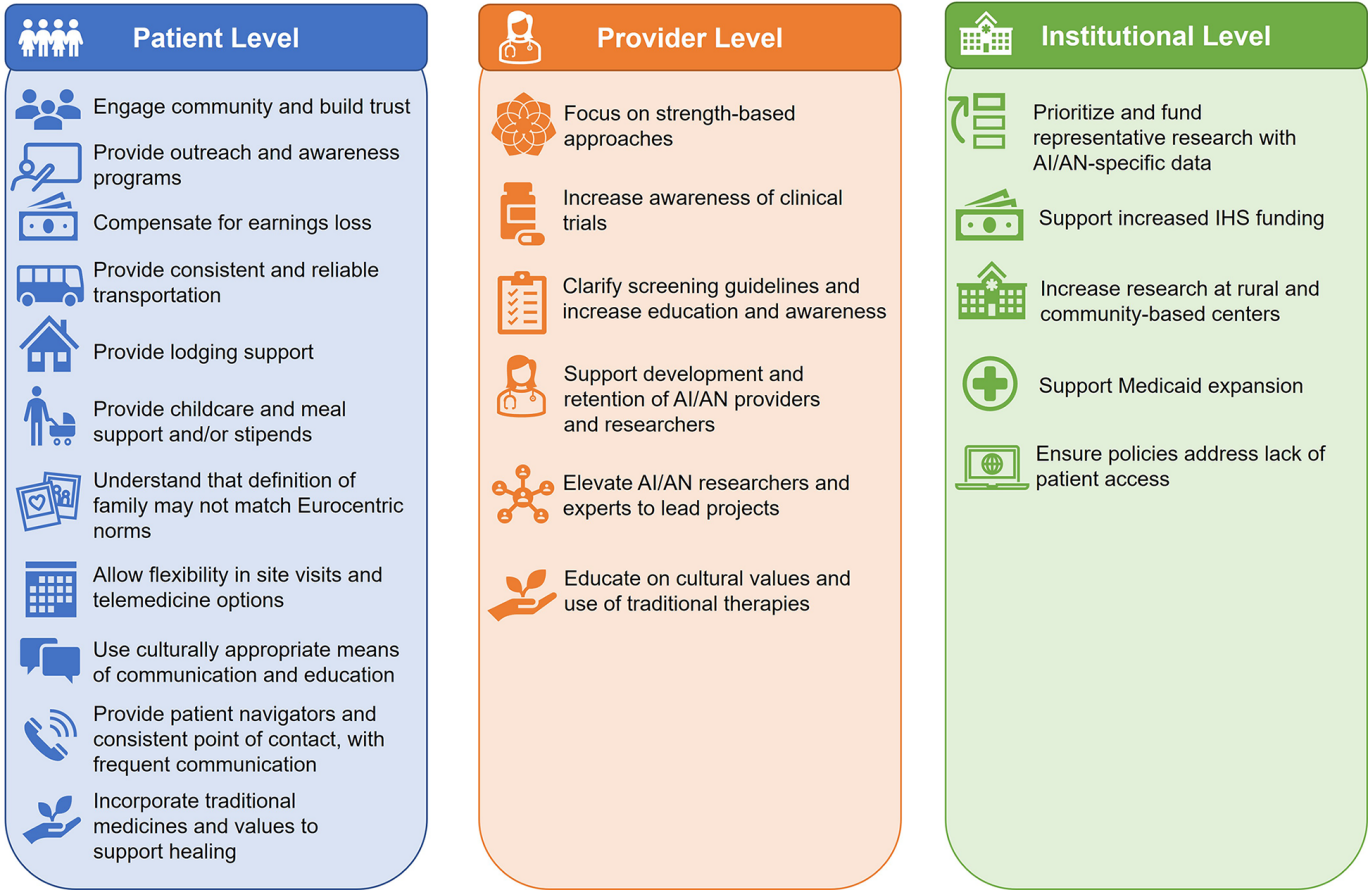
Best practices for decreasing barriers to care for AI/AN individuals. AI/AN, American Indian and Alaska Native; IHS, Indian Health Service.

The pharmaceutical industry has the opportunity to partner with AI/AN researchers and providers as well as community leaders to alleviate healthcare and clinical trial barriers at the patient, provider, and institutional levels to support equitable access to care for AI/AN individuals (Figure 4). AI/AN investigators, researchers, and support staff should be provided with the opportunity to actively lead and participate in research efforts. Advisory boards are one tool the pharmaceutical industry can use to partner with AI/AN healthcare professionals to gain valuable insights and guide steps to increase access to healthcare more broadly and through increased clinical trial enrollment for AI/AN individuals. Furthermore, AI/AN doctors are more likely than non-AI/AN peers to serve AI/AN patients (108) and, therefore, support efforts to increase enrollment of AI/AN individuals in medical school along with other public health and PhD programs to build a workforce with expertise in Indigenous health issues, which is essential (109).

### Addressing institutional-level barriers

Health disparities in AI/AN communities are likely to remain unless there is improved funding, streamlined services, and increased workforce talent in IHS and other healthcare systems. Eliminating administrative barriers and facilitating and investing in partnerships between IHS, Tribal Nations, and surrounding non-Indigenous healthcare facilities could improve care by providing coordinated, culturally competent, and qualified care at all levels.

Medicaid plays a meaningful part in providing health services for AI/AN peoples (63, 110). In 2018, 1.8 million AI/AN individuals were covered by Medicaid, including 36% of adults under 65 years (111). Medicaid expansion is vital to improving access to equitable care and clinical trial involvement for AI/AN individuals. As of January 1, 2022, the Clinical Treatment Act requires Medicaid to cover the clinical care costs associated with participation in qualifying clinical trials, which may remove some barriers to participation by AI/AN individuals (112). In addition, expanding Medicaid in states that have not yet done so may also increase the number of AI/AN patients who could participate in clinical trials.

## A path forward

Representative clinical research that generalizes to a real-world population is essential for advancing health equity and fostering trust in the safety and efficacy of therapies. However, efforts to increase enrollment of AI/AN individuals in clinical research need to be done in an ethically, culturally responsive, and responsible manner (2). AI/AN individuals should have an adequate understanding of study participation benefits and risks prior to enrollment. Studies that are more accessible to members of AI/AN communities and those that potentially involve topics of concern to the greater community have been able to recruit the highest percentage of AI/AN participants (2). Understanding the societal and environmental contributors to high rates of cancer in AI/AN populations and the specific outcomes of treatments in AI/AN groups based on their geographic region is also vital for health equity moving forward.

### Examples of inclusive research

The CATORI trial (NCT05624788) is an observational study to define care pathways of AI/AN patients requiring specialty care (neurologist, ophthalmologist, or oncologist) and evaluate the feasibility of clinical research in the existing structure (113). The primary outcome is the percentage of participants seen by a specialist for advanced care after primary care provider referral to a specialist at 6 and 12 months. Patient barriers to specialty referral will be collected as a secondary outcome.

Two clinical trials, EMPACTA and ELEVATUM, were designed to highlight underrepresented populations in the US (114, 115). The Phase III EMPACTA trial (NCT04372186) in patients with COVID-19 pneumonia intentionally included underrepresented patients and addressed inequitable healthcare access for patients who were at high risk with substantial unmet needs and/or racial and ethnic minorities (114). Of the 337 patients enrolled, 13% were AI/AN, 15% were Black, and 56% were Hispanic or Latino (114). EMPACTA demonstrated that prioritizing the enrollment of participants from historically underrepresented racial and ethnic groups can improve patient access to therapies and advance scientific knowledge. ELEVATUM (NCT05224102), a post-approval study in patients with diabetic macular edema who self-identify as Black/African American, Hispanic/Latino, Native American/AN/Native Hawaiian or other Pacific Islander, used best practices from EMPACTA to remove participation barriers for historically underrepresented patients (115). This included expanding the network of clinical trial sites to include investigators who treat diverse patient populations and expanding inclusion criteria and logistical support.

The Walking Forward program was established in 2002 to address the high cancer mortality rates observed in the AI population in western South Dakota (116, 117). Over 4,000 AI individuals have participated in the research program (117), and 30 manuscripts have been published describing processes and outcomes of the Walking Forward program, which has shown that improving cultural competency in cancer care through the incorporation of patient navigators can result in trusting partnerships, improve cancer screening rates, and increase patient adherence to radiation therapy (118).

While these examples are a step toward health equity, more needs to be done. The six overarching recommendations of the American Society of Clinical Oncology and Association of Community Cancer Centers joint call for increased racial and ethnic diversity in cancer clinical trials fall into distinct themes that echo much of what we have stated in the current review: partnership with community leaders; enhanced provider-patient education; and increased investment in programs, policies, and infrastructure (78). These recommendations along with those in the cardiovascular, dermatology, and respiratory fields of research highlight the growing awareness and need for increasing inclusiveness (119–121).

## Conclusions

An emphasis on a strengths-based approach that incorporates the AI/AN lens may help to address barriers perpetuated in the healthcare system that influence access to high-quality specialty care. Community-based partnerships and strength- and trust-based approaches are essential tools to promote equitable access to high-quality specialty care. Further, these partnerships are likely to foster participation of AI/AN individuals and communities in clinical research. They may also increase access to healthy foods and opportunities for AI/AN communities to become more active and mobile and develop sustainable healthy habits. With the tremendous diversity of AI/AN populations, including unique histories, cultures, and languages, multiple approaches should be used to decrease barriers to healthcare and clinical research because what works for one Tribal Nation may not have the same benefit in another Tribal Nation. It is also important to measure the impact of efforts to decrease barriers to care and continually obtain feedback from the community and its leaders.

Those committed to improving access for AI/AN communities should seek opportunities to listen, learn, and co-create solutions to address existing barriers. One way this can be accomplished is through educational materials that allow providers to understand traditional practices such as AI/AN traditional medicines and their importance in Tribal cultures as well as their roots in modern, pharmaceutical compounds. By working together, the pharmaceutical industry, healthcare providers, researchers, Tribal leaders, and other key stakeholders can begin breaking down the barriers to health equity experienced by AI/AN communities.
